# Long-term passive acoustic recordings track the changing distribution of North Atlantic right whales (*Eubalaena glacialis*) from 2004 to 2014

**DOI:** 10.1038/s41598-017-13359-3

**Published:** 2017-10-18

**Authors:** Genevieve E. Davis, Mark F. Baumgartner, Julianne M. Bonnell, Joel Bell, Catherine Berchok, Jacqueline Bort Thornton, Solange Brault, Gary Buchanan, Russell A. Charif, Danielle Cholewiak, Christopher W. Clark, Peter Corkeron, Julien Delarue, Kathleen Dudzinski, Leila Hatch, John Hildebrand, Lynne Hodge, Holger Klinck, Scott Kraus, Bruce Martin, David K. Mellinger, Hilary Moors-Murphy, Sharon Nieukirk, Douglas P. Nowacek, Susan Parks, Andrew J. Read, Aaron N. Rice, Denise Risch, Ana Širović, Melissa Soldevilla, Kate Stafford, Joy E. Stanistreet, Erin Summers, Sean Todd, Ann Warde, Sofie M. Van Parijs

**Affiliations:** 1 NOAA Northeast Fisheries Science Center, 166 Water Street, Woods Hole, MA 02543 USA; 2 University of Massachusetts Boston, 100 Morrissey Blvd, Boston, MA 02125 USA; 3 Woods Hole Oceanographic Institution, 266 Woods Hole Road, Woods Hole, MA 02543 USA; 4 Naval Facilities Engineering Command Atlantic, Norfolk, VA 23508 USA; 5 NOAA Alaska Fisheries Science Center, 7600 Sand Point Way N.E., Seattle, WA 98115 USA; 6 New Jersey Department of Environmental Protection, Trenton, NJ 08625 USA; 7 Bioacoustics Research Program, Cornell Lab of Ornithology, Cornell University, 159 Sapsucker Woods Road, Ithaca, NY 14850 USA; 8 JASCO Applied Sciences, 32 Troop Ave, Dartmouth, NS B3B 1Z1 Canada; 9 Dolphin Communication Project, Port St. Lucie, FL 34985 USA; 10 NOAA Stellwagen Bank National Marine Sanctuary, 175 Edward Foster Road, Scituate, MA 02066 USA; 11 Scripps Institution of Oceanography, University of California San Diego, 9500 Gilman Drive MC 0205, La Jolla, CA 92037 USA; 12 Nicholas School of the Environment, Duke University Marine Laboratory, 135 Duke Marine Lab Road, Beaufort, NC 28516 USA; 13 Oregon State University, 2030 SE Marine Science Drive, Newport, OR 97365 USA; 14 New England Aquarium, Central Wharf, Boston, MA 02110 USA; 15 Fisheries and Oceans Canada, Bedford Institute of Oceanography, 1 Challenger Drive, Dartmouth, NS B2Y 4A2 Canada; 16 Syracuse University, 107 College Place, Syracuse, NY 13244 USA; 17 The Scottish Association for Marine Science (SAMS), Oban, PA37 1QA Scotland UK; 18 NOAA Southeast Fisheries Science Center, 75 Virginia Beach Drive, Miami, FL 33149 USA; 19 University of Washington, Applied Physics Laboratory, 1013 NE 40th Street, Seattle, WA 98105 USA; 20 Maine Department of Marine Resources, West Boothbay Harbor, ME 04575 USA; 21 Allied Whale, College of the Atlantic, 105 Eden Street, Bar Harbor, ME 04609 USA; 22 Pratt School of Engineering, Duke University, Durham, NC 27708 USA

## Abstract

Given new distribution patterns of the endangered North Atlantic right whale (NARW; *Eubalaena glacialis*) population in recent years, an improved understanding of spatio-temporal movements are imperative for the conservation of this species. While so far visual data have provided most information on NARW movements, passive acoustic monitoring (PAM) was used in this study in order to better capture year-round NARW presence. This project used PAM data from 2004 to 2014 collected by 19 organizations throughout the western North Atlantic Ocean. Overall, data from 324 recorders (35,600 days) were processed and analyzed using a classification and detection system. Results highlight almost year-round habitat use of the western North Atlantic Ocean, with a decrease in detections in waters off Cape Hatteras, North Carolina in summer and fall. Data collected post 2010 showed an increased NARW presence in the mid-Atlantic region and a simultaneous decrease in the northern Gulf of Maine. In addition, NARWs were widely distributed across most regions throughout winter months. This study demonstrates that a large-scale analysis of PAM data provides significant value to understanding and tracking shifts in large whale movements over long time scales.

## Introduction

Understanding the distribution and movement patterns of marine mammals is essential for supporting their conservation^1,2^. Large whales undertake some of the longest of mammalian migrations, with some species traveling over 10,000 kilometers annually^3^. Several species of baleen whales are known to migrate from productive feeding grounds at higher latitudes in the summer to winter breeding grounds at lower latitudes^4^, although not all individuals leave high latitudes each year^5,6^. The selective pressures driving these movements remain unresolved, including the relative importance of environmental influences and predator avoidance^7–9^. Some species, such as humpback (*Megaptera novaeangliae*) and gray (*Eschrichtius robustus*) whales, have relatively well-documented migration routes to and from known wintering grounds^10,11^. The migratory movements of other species, for example North Atlantic minke whales (*Balaenoptera acutorostrata*), have only been described recently^12^, while those of others, like Antarctic blue whales (*B*. *musculus intermedia*), remain poorly known^13^.

North Atlantic right whales (NARW; *Eubalaena glacialis*) are one of the least abundant, but intensively studied, species of baleen whales^14^. Centuries of whaling resulted in their near extinction by the time protection was introduced in 1935^15^. Their abundance increased from approximately 350 in 1990^16^ to 476 in 2010^17^, but has since shown evidence of decline^18^. The reasons for this decline are still unknown, but both widespread anthropogenic impacts from fishing and shipping, and climatic changes are likely. Much of the substantial research program that informs NARW conservation efforts is managed through the North Atlantic Right Whale Consortium (NARWC) formed in 1986. The NARWC curates an extensive catalog of geo-referenced photo identifications of individual whales, coupled with genetic, fisheries entanglement, and health-related data, that provides a detailed understanding of the status of the individuals comprising this species^19^.

These data come primarily from decades of research that has been focused in areas that NARWs are known to use, including winter calving grounds off the coasts of Florida and Georgia, and more northerly feeding grounds off the coasts of New England and Atlantic Canada^15^. Although the general distribution of NARWs seemed fairly well known, recent surprises included discovering a potential mating ground in the Gulf of Maine in the winter^20,21^; acoustic detections and visual observations of whales in their historically-recorded habitats off Greenland and Iceland^15,22^; or year-round presence in locations previously thought of as migratory corridors^23–25^. Sporadic sightings of solitary NARWs in European waters^26–28^, such as the 131 day round-trip made by one individual from U.S. waters to an old whaling ground off northern Norway^29^, demonstrate that our understanding of their movement patterns remains incomplete.

Management to mitigate impacts of human threats to NARWs includes spatio-temporal measures, such as Seasonal Management Areas (SMAs) or Areas to be Avoided (ATBA), within which ships are required (SMA) or encouraged (ATBA) to reduce speed or avoid altogether during specified times when whales are known to use these areas^30,31^. For this reason, it is important to gain a better understanding of the seasonal distribution of NARWs, including how this distribution has changed in recent years. Since 2010, there has been a noticeable shift in the distribution of NARWs. Whales appear to visit some regions much less frequently, such as the Bay of Fundy, where they were regularly observed for at least two decades^32^. Likewise, there have been recent years with substantially reduced sightings of NARWs in the broader Gulf of Maine^33^. On the other hand, the proportion of the population that uses Cape Cod Bay, in the southern Gulf of Maine, appears to have increased as of late^34^.

Until the early 2000s, data collection on NARWs mainly consisted of visual effort from ship-based or aerial surveys^35^. Visual surveys are limited by daylight, weather conditions, and the availability of suitable research platforms at appropriate times and in appropriate locations^36^. Emerging technologies, such as PAM, provide new ways to survey large areas. PAM can provide (1) continuous coverage of areas that are otherwise hard to observe for species presence, (2) data on multiple species simultaneously, and (3) information on the acoustic habitat, including natural and anthropogenic sounds^36,37^. Passive acoustic recorders deployed at different locations and over multiple years can comprise a novel, persistent, large-scale monitoring network that is impossible to replicate with other technologies. Such a network can provide detailed long-term information on multiple species to help inform management^38,39^.

There have been numerous deployments of passive acoustic recorders along the eastern seaboard of North America from Florida, USA to Nunavut, Canada^25,40–42^. These deployments were designed to answer a variety of different individual research and management questions, but in combination, these data provide substantial coverage of the continental shelves off the western North Atlantic Ocean since 2004. A collaborative program across multiple institutions has brought these data together to assess the holistic occurrence of calling NARWs throughout this area. Here, we show how these acoustic data demonstrate changes in the distribution of NARWs across the eastern seaboard of North America during the past decade (2004–2014).

## Results

**Figure 1:**
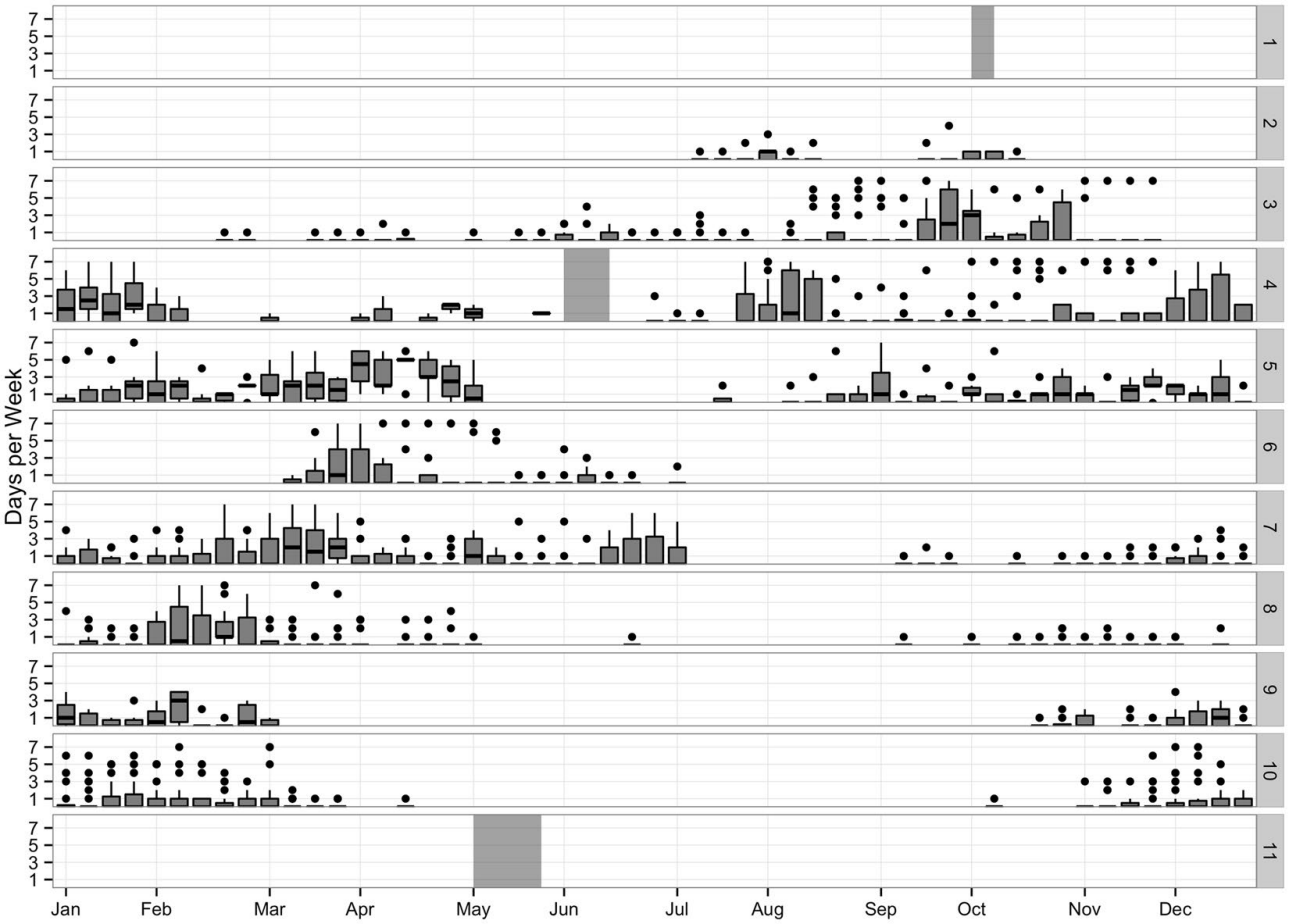
Weekly Presence Summary: Boxplots representing the number of days per calendar week with confirmed North Atlantic right whale upcall acoustic presence in each region described in Fig. 4 and for all years of the study (2004–2014). Horizontal lines within the boxes indicate the median, box boundaries indicate the 25^th^ (lower boundary) and 75^th^ (upper boundary) percentiles, vertical lines indicate minimum and maximum values, and black dots represent outliers. Grey blocks indicate weeks where no data were available for that region.

A total of 35,600 days of acoustic recordings were processed with the Low Frequency Detection and Classification System (LFDCS)^43^, and subsequent NARW upcall detections were manually reviewed, resulting in 2,527 days (7%) with confirmed NARW acoustic presence. NARWs were acoustically present along the entire eastern seaboard of North America from the western Scotian Shelf (region 3) to the waters off Jacksonville, Florida (region 10) throughout the winter months from late October through early April, with the exception of no detections on the Scotian Shelf from December through February (Fig. 1). A decrease in detections was seen in summer months in southern regions, reflecting the known movement of breeding individuals towards northern feeding grounds. NARWs were also detected near Iceland and Greenland (region 2) from July-October (see previous analysis by Mellinger, *et al*.^22^). Davis Strait (region 1) contained one day with possible NARW detections in both December and March. However, with high bowhead whale (*Balaena mysticetus*) and humpback whale calling also present, NARW presence could not be confirmed with confidence. There were no NARW detections in any recordings from Bermuda or the Caribbean (region 11), confirming the likelihood that NARWs do not currently venture into the waters surrounding Bermuda and the Caribbean.

**Figure 2:**
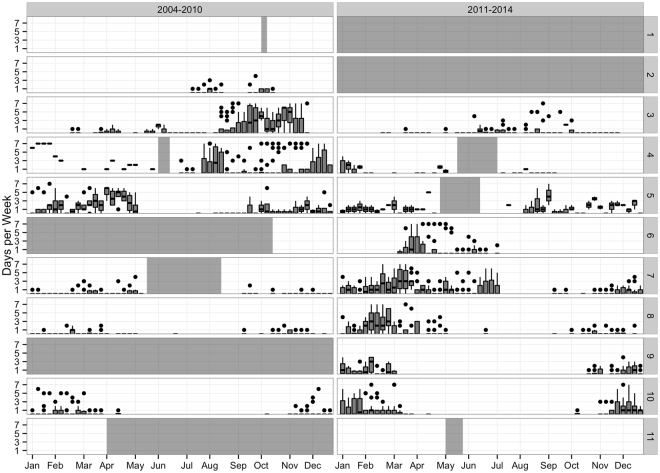
Weekly Presence Comparison from 2004–2014: Boxplots representing the number of days per calendar week with confirmed North Atlantic right whale upcall acoustic presence in each region described in Fig. 4 and for each time period of interest (2004–2010 and 2011–2014). Horizontal lines within the boxes indicate the median, box boundaries indicate the 25^th^ (lower boundary) and 75^th^ (upper boundary) percentiles, vertical lines indicate minimum and maximum values, and black dots represent outliers. Grey blocks indicate time periods where no data were available for that region.

Average weekly acoustic presence was broken up into two time periods representing before (2004–2010) and after (2011–2014) the described distribution shift starting in 2010 (Fig. 2)^33^. To test whether the occurrence of right whales in regions differed over the two time periods, we ran a Generalized Linear Model (GLM; see methods) with the number of days in which whale calls were detected as the dependent variable, and the time periods (2004–2010; 2011–2014) and regions as independent variables, with their interaction effects included in the model. There were too few data (in some time*region cells) available for Davis Strait, Iceland and Greenland, Georges Bank, Cape Hatteras, and Bermuda and the Caribbean (regions 1, 2, 6, 9 and 11) for them to be included in the model.

For all other regions, both factors and their interactions were significant (Table 1). Pairwise comparisons of time periods across individual regions (run using phia::testInteractions) demonstrated differences between the two time periods (Table 2), except for in Massachusetts Bay (region 5). Northern regions (3 and 4; Scotian Shelf and Gulf of Maine) saw a reduction in calls in 2011–2014, but mid-Atlantic regions (7 and 8; southern New England and the mid-Atlantic), and the Southeastern U.S. (region 10) saw increases (Table 3, produced using phia:interactionMeans). We used the False Discovery Rate^44^ to adjust for alpha-value inflation.

**Table 1:** Results of the Poisson Generalized Linear Model (GLM) testing whether the occurrence of North Atlantic right whales in the regions differed over two time periods (2004–2010; 2011–2014).

Response: # days with NARW presence	Chi-Square	Degrees of Freedom	P-value
timePeriod	21.57	1	3.41e-06***
Region	542.81	5	<2.2e-16***
timePeriod:Region	489.70	5	<2.2e-16***

The number of days in which whale calls were detected is the dependent variable, and the time periods and regions are independent variables, with their interaction effects included in the model. Davis Strait, Iceland and Greenland, Georges Bank, Cape Hatteras, and Bermuda and the Caribbean (regions 1, 2, 6, 9 and 11) are excluded from the model due to insufficient data in some time*region cells. For all other regions, both factors and their interactions were significant. Table 1 shows the overall GLM results between the two time periods (timePeriod) and region (Region). (***) indicates significance of P < 0.001.

**Table 2:** Results from the Poisson Generalized Linear Model (GLM) testing between the two time periods (A–B) for each region separately, using the False Discovery Rate^44^ to correct for alpha-value inflation.

Region	Value	Degrees of Freedom	Chi-Square	P-value
A-B: 3	5.07	1	136.43	<2.2e-16***
A-B: 4	3.19	1	28.31	1.24e-07***
A-B: 5	1.03	1	0.07	0.78
A-B: 7	0.18	1	99.70	<2.2e-16***
A-B: 8	0.18	1	59.67	2.25e-14***
A-B: 10	0.46	1	42.21	1.23e-10***
Residuals: 42				

Pairwise comparisons of time periods across individual regions were run using phia::testInteractions^69^. (***) indicates significance of P < 0.001.

**Table 3:** Values for each region and time period individually from the Poisson Generalized Linear Model (GLM).

Time Period	Region	Adjusted Mean	Standard Error of Link
2004–2010	3	18.89	0.07
2011–2014	3	3.73	0.12
2004–2010	4	20.85	0.05
2011–2014	4	6.53	0.21
2004–2010	5	20.71	0.07
2011–2014	5	20.06	0.09
2004–2010	7	2.12	0.17
2011–2014	7	12.06	0.04
2004–2010	8	1.18	0.21
2011–2014	8	6.40	0.07
2004–2010	10	4.81	0.10
2011–2014	10	10.40	0.06

Results were produced using phia:interactionMeans^69^.

**Figure 3:**
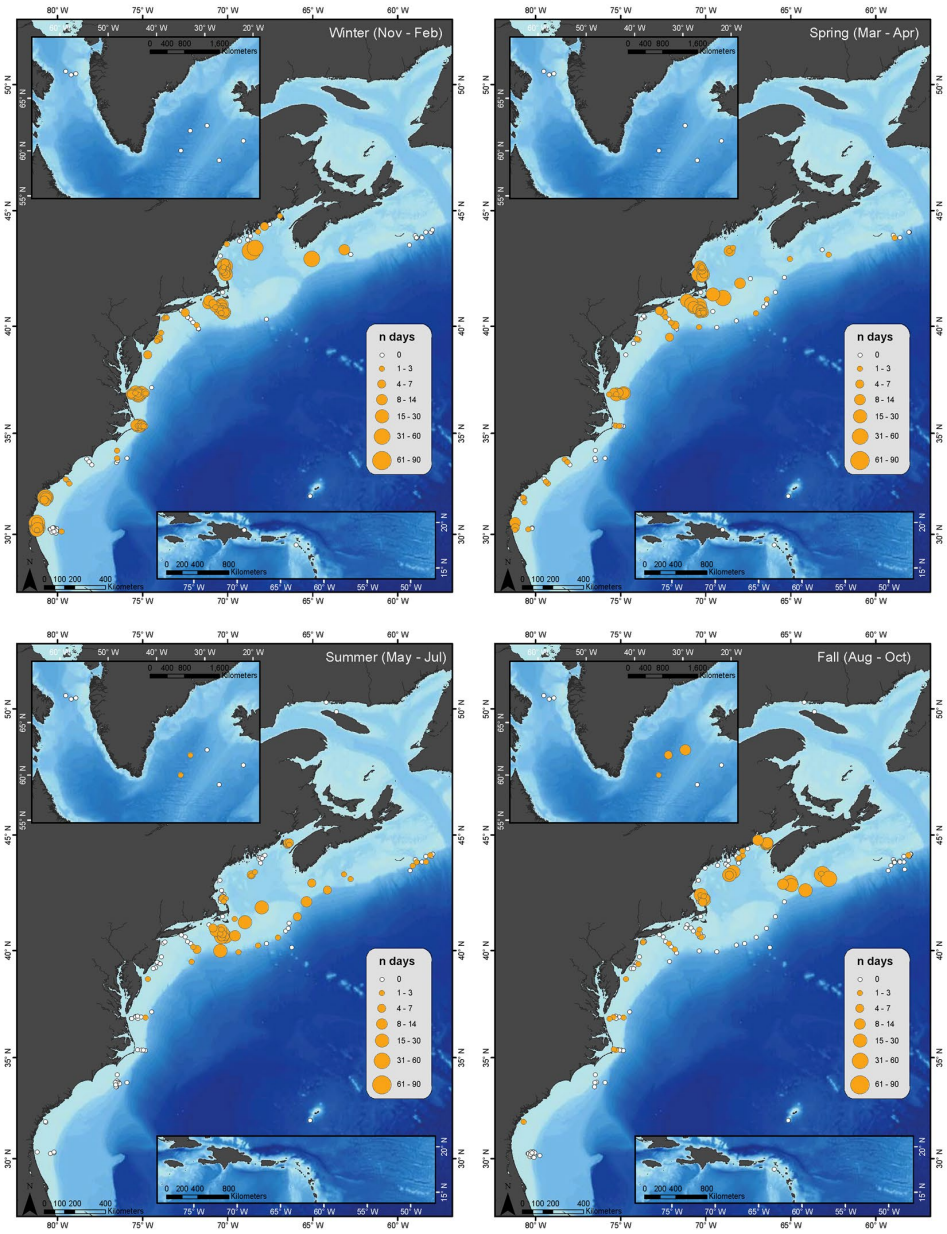
Seasonal Occurrence Maps: The number of days per season with confirmed North Atlantic right whale (NARW) upcall acoustic detections, summarized for all available recordings locations (2004–2014). Filled orange circles indicate NARW acoustic presence, and circle size indicates the number of days with NARW acoustic detections during a season. White dots indicate recorder locations with no NARW acoustic presence for any year during that season. Figure produced with ArcGIS 10.3.1 (http://www.arcgis.com); background map credits: Esri and GEBCO.

Spatial distribution of NARW acoustic occurrence was summarized from 2004–2014 by seasons in Fig. 3. Seasons were defined based on Roberts, *et al*.^45^ with November to February as Winter, March to April as Spring, May to July as Summer, and August to October as Fall. Seasonal acoustic occurrence of NARWs reflected patterns seen in the daily presence plots (Figs 1 and 2), with NARW presence along the entire coast in both winter and spring seasons. Across all seasons, NARWs were detected from Cape Hatteras (region 9) to Nova Scotia (region 3), highlighting the expansive habitat of NARWs for most of the year (Fig. 3).

The LFDCS, with a Mahalanobis Distance (MD) threshold of 3.0, missed days with NARW upcalls at an estimated rate of 31%; if we used a threshold of one true upcall detection per day for daily presence, the rate of missed days with NARW upcalls would have been 25%. Manually tallied upcalls from the Gulf of Maine analysis^21^ revealed that days where LFDCS detected true NARW upcalls had a median of 259 calls per day and ranged from 20–2770 calls per day, and days where LFDCS missed NARW upcalls had a median of 7 calls per day and ranged from 1–66 calls per day. It is important to note that detection rates, and therefore missed detection rates, will be highly variable due to variability in ambient noise levels, recorder types, habitat, bathymetry, and calling behavior of the animals. However, this analysis indicates that at greater calling rates, indicative of higher calling activity and potentially reflective of multiple calling individuals, daily NARW acoustic presence was most likely captured in areas where they were vocalizing. Sporadic calls, which may be produced by lone individuals passing through an area, were less likely to be detected in this analysis (note that lone animals can be missed by visual surveys, particularly aerial surveys, as well). Given the main goals of capturing broad-scale movements of the entire population with this analysis, failing to detect the presence of some individuals does not compromise the overall results.

## Discussion

This study on acoustic presence of NARWs demonstrates this species’ high mobility and broad geographic range. It also shows that the habitats NARWs frequent can change over time and that they are often distributed widely across their entire range. Previous understanding of NARW movements assumed the majority of the population migrated between the calving grounds in winter months and northern feeding grounds in summer months, where visual survey effort was therefore concentrated^27,46^. Little was known about the whereabouts of whales that did not frequent these habitats, or the exact timing and location of other important areas. This study demonstrates nearly continuous year-round NARW presence across their entire habitat range, particularly north of Cape Hatteras, suggesting that not all of the population undergoes a consistent annual migration. It is possible that the non-migrating whales could be mobile individuals occupying broader areas throughout the year, like some subspecies of blue whales in the Antarctic^47^, or individuals that do not migrate annually, like a portion of the east Australian population of humpback whales^5^. Our data clearly demonstrate that NARWs occur along the entire eastern seaboard of North America for most of the year, even if that distribution has shifted within the past decade.

The acoustic data supports NARW distributional changes that have been recently observed during visual surveys. Furthermore, the acoustic data provide additional insights into where NARWs are located at times of the year when the poor weather and lack of light make visual surveys highly restricted (i.e., late fall to early spring). NARWs appear to have shifted from previously prevalent northern grounds, such as the Bay of Fundy and greater Gulf of Maine (regions 3 and 4), to spending more time in mid-Atlantic regions year-round (regions 7 and 8)^25,48^. In addition, improved visual survey effort in Canadian waters shows that NARWs have also been sighted more frequently further north in areas such as the Gulf of Saint Lawrence^35^. Acoustic data are currently being processed to allow further evaluation of this region, but were not available for this study. This is an area of extreme importance, with at least 10 linked NARW deaths this year alone^49^. Moving forward, it is crucial to have better available coverage for past and future monitoring efforts in such important regions where entanglement and ship strike threats are prominent. Likewise, we know of past NARW occurrence in historical habitat areas such as Greenland and Iceland, with acoustic presence in 2007^22^, and it would be valuable to understand current usage of this region and confirm the northern extent of their range with more recent data. Therefore, this type of long-term, large-scale approach using PAM is invaluable for helping to track presence, and changes in presence, of key species to understand (1) how they have shifted their distribution and (2) whether these shifts puts them at increased risk from anthropogenic threats.

It remains unclear if these observed distribution shifts are due to environmental or anthropogenic effects, if they are a response to short-term changes in the environment, or part of a longer-term cycle in which NARWs shift their distribution. With recent studies finding the Gulf of Maine is the fastest warming body of water in the world^50^, it is not surprising to see distributional changes across marine species. We suspect further changes in distributions will occur as water temperatures continue to rise, forcing movements towards both favorable oceanographic conditions and food sources elsewhere. Regardless of the factors influencing these changes in distribution, it is critical for management strategies to reflect new threats that may arise for this species as they move into regions outside of existing management areas^51^.

The purpose of this study was to provide baseline information of NARW distributions across their current range. Here, PAM data was used for distributional analysis and assumes homogeneity in the probability of upcalls across all regions. We caution that this is not necessarily the case, as known calling rates vary among demographic groups, such as quieter mother-calf pairs^52^, resulting in lower detection probabilities. Call rates are also lower with certain behaviors, such as foraging and logging^53^, and therefore it is reasonable to suspect different calling rates over the different habitats and seasons summarized in this study. Additional variation exists across all the regions, with detection probabilities likely varying with different acoustic habitats, bathymetry and recorders. Risch, *et al*.^12^ show that ambient noise levels varied across three sites included in this study (Massachusetts Bay, mid-Atlantic, and Southeastern U.S.; regions 5, 7, and 10) over each season, affecting detection probabilities and ranges; while Rice, *et al*.^54^ provide detailed results on the varying acoustic environment at 10 different sites within the NARW range. These studies highlight the challenges with large-scale datasets, however, further analysis into detection ranges were beyond the scope and goal of this study. Thus, this work used an average range of detection distances based on published studies^55,56^. As such, it provides a minimum estimate of NARW presence, with the understanding that in some regions the detection ranges may have been slightly more constricted or wider than the average used.

This study integrates data from a suite of smaller-scale studies focused on the fine-scale occurrence of NARWs and other species. Comparing our results with these smaller-scale studies such as Hodge, *et al*.^48^, Bort, *et al*.^21^, Salisbury, *et al*.^25^, Mellinger, *et al*.^22^, Morano, *et al*.^23^, and Whitt, *et al*.^57^, there are time periods with discrepancies between the NARW acoustic detections presented in our study and these previous studies, with the previous studies finding right whale calls in some months that we did not find confirmed detections. This is likely due to a difference in detectors and analysis methodology. In order for this study to include and process such a large acoustic dataset, it was necessary to use an automated detector, LFDCS, and manually review these detections at a coarser level than may have been done in the smaller-scale studies. Our review of the LFDCS performance with a MD threshold of 3.0 at three sites found that 31% of days with right whales present were missed when compared to a full manual evaluation of the data, and that days with lower calling rates tended to be missed more often. Therefore, our results represent a minimum presence compared with these more detailed studies, and it is possible that NARWs have higher occurrence in some areas than is reported here. The missed detection rate could be reduced by lowering our classification threshold (i.e., increasing the maximum MD), but at the cost of increasing the time required to manually screen each detection, since increasing the maximum MD will result in more detections that must be reviewed by an analyst. As with all detection systems, there is a balance between accuracy and processing time that must be considered when choosing a detector within the context and scope of any study.

This is one of the first comprehensive, long-term passive acoustic studies to investigate an entire habitat range for a marine mammal at this temporal and spatial scale, and is made possible only by the cooperation and collaboration of an extensive research community. Even in areas where data were collected for alternate purposes, the combined contributed recordings provided crucial information to assess both the acoustic occurrence and changing distribution of the NARW population. This analysis demonstrates what can be accomplished for other poorly understood species, and encourages broad research collaborations in the future. All contributing data sets were combined for the common goal of understanding the distribution of a critically endangered species facing extreme threats from anthropogenic and environmental influences. In planning future large-scale studies, standardization of acoustic recorders and methods should be considered to improve the quality of datasets.

PAM is a powerful, cost-effective, long-term monitoring tool that can give a better understanding of temporal trends and reveal range expansion, decline, or distribution shifts in populations, as well as interannual changes. This information can be used to direct science and management to focal areas of interest. Most importantly, in an ocean where conditions are changing rapidly, adaptive management is needed to identify and protect areas that are crucial for species on the brink of extinction. Potential ways forward include setting up real-time passive acoustic monitoring systems (see NEPAN^39^), or thinking beyond the traditional means of classifying NARW critical habitat as static, confined areas. This is especially relevant when considering the mobile nature of this species whose distribution patterns may still be changing. It is imperative to continue effective surveys and timely conservation efforts to ensure the recovery of this endangered species.

## Methods

### Data collection

**Figure 4:**
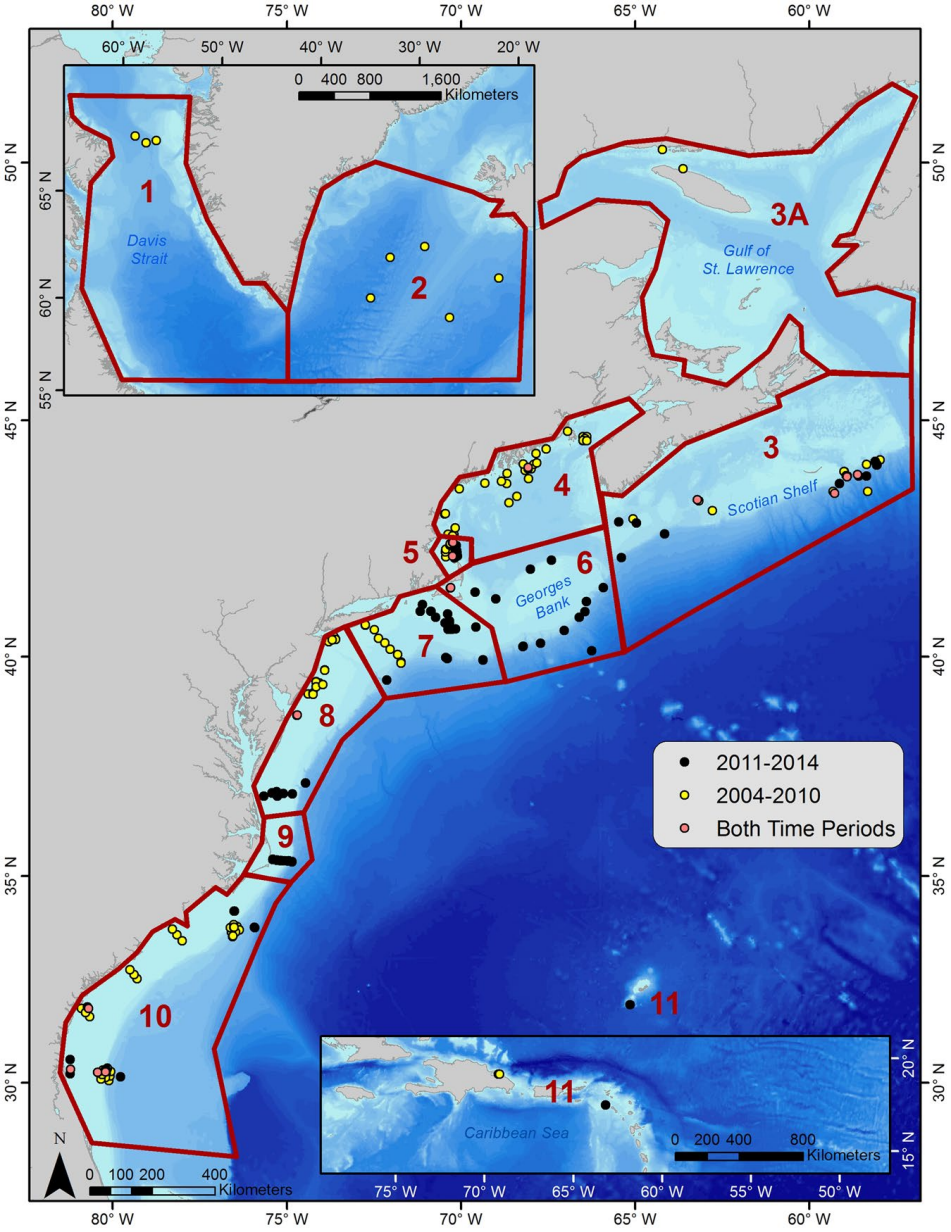
Recorder Locations: Locations of available passive acoustic recorders used for this study spanning from Bermuda and the Caribbean (bottom right map inset) to the northernmost locations in Davis Straight (top left map inset). Yellow points indicate the locations of recorders available from 2004–2010; black points indicate the locations of recorders available from 2011–2014; and pink points indicate locations of recorders available for both time periods. Red boundaries outline the designated regions, which were defined by the historical distribution patterns of North Atlantic right whales across their range. Region numbers correspond to the following geographic areas: 1. Davis Strait; 2. Iceland and Greenland; 3. Scotian Shelf; 4. Northern Gulf of Maine; 5. Massachusetts Bay; 6. Georges Bank; 7. Southern New England; 8. Mid-Atlantic; 9. Cape Hatteras; 10. Southeastern U.S.; 11. Bermuda and the Caribbean. Figure produced with ArcGIS 10.3.1 (http://www.arcgis.com); background map credits: Esri and GEBCO.

**Figure 5:**
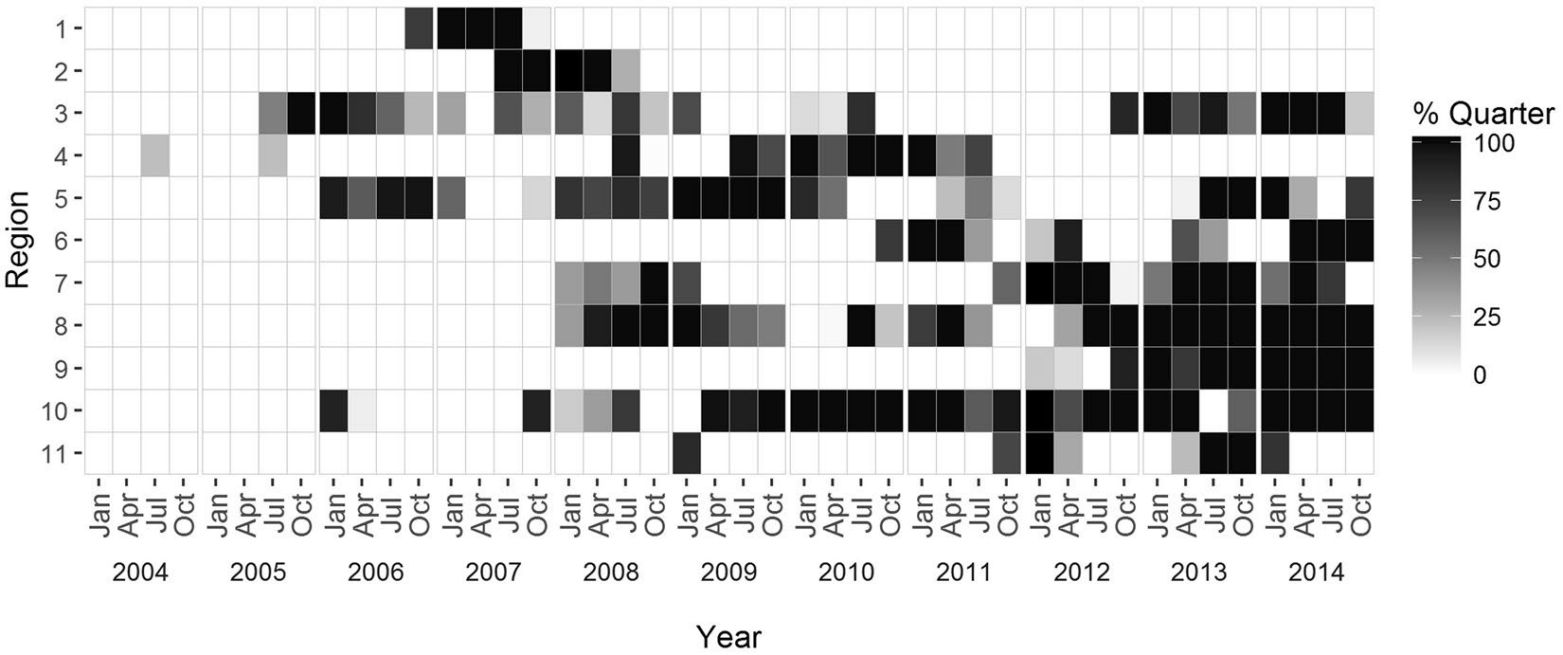
Recording Effort: Figure indicating the proportion of year with available passive acoustic recordings in each region (see Fig. 1). Years are split into quarters from January 2004 to December 2014. Black indicates at least one recorder present for the entire quarter year for that region, lighter gray indicates a portion of that time period with recordings, and white indicates no available acoustic data for that region and time period.

Passive acoustic data collected for a multitude of different research projects and goals were combined to examine a decade-long acoustic record of the spatial and temporal occurrence of NARWs throughout the western North Atlantic Ocean. This large area was divided into 11 regions, covering the main historical areas where the existing NARW population has been found since the late 1600s, ranging from Florida, USA to Cape Farewell, Greenland^58^. Recorders were assigned a region based on the biological and geographical importance of the area (Fig. 4). Region 3 was broken up to include subregion 3A, representing the Gulf of St. Lawrence: an area having an increase in NARW sightings and research effort over the last few years, but insufficient acoustic data available to contribute to this study. Data were examined across two time periods (2004–2010 and 2011–2014) representing before and after the observed distribution shift in 2010. Due to the ad hoc nature of this large-scale collaborative project, available data are patchy, a few recorders were duty cycled, and there are some regions or time-periods with no available data (Fig. 5).

Five types of bottom-mounted passive acoustic recorders were deployed from 2004 through 2014 (See Supplementary Table 1): the High-frequency Acoustic Recording Package (HARP)^59^, the Marine Autonomous Recording Unit (MARU)^55^, the Autonomous Multichannel Acoustic Recorder (AMAR)^60^, the National Oceanic and Atmospheric Administration’s (NOAA) Pacific Marine Environmental Laboratory’s (PMEL) Moored Autonomous Hydrophones (HARU)^61^, and the Guardbuoy (http://geospectrum.ca/guard-buoy). Data collected from each of these recorders varied from a minimum of 25 days to a maximum of 2 years (Supplementary Table 1). Some recorders (59 out of 324) were duty cycled, ranging in recording from 12–95% of the time, while most (265 out of 324) recorded continuously. The spatial configuration in which they were deployed varied from single units to lines and arrays of recorders; the configuration was determined by the original goal of the specific research project in question (Fig. 4). The majority of recordings were sampled at 2 kHz, with some ranging up to 250 kHz. All recordings were low-pass filtered and decimated to 2 kHz for analytical consistency across data in order to make them comparable.

Maximum detection ranges for NARWs can vary considerably, depending on recording equipment, location, and environmental conditions, as well as call type and behavioral context^62^, but are estimated to range from 8 to 16 km^55,56^. Consequently, single recorders were selected for analysis from array configurations with units spaced less than 8 km apart, to ensure full coverage of the area while minimizing duplicative detections across recorders. We focused our analyses on data collected between January 2006 to December 2014, with the exception of data collected in 2004 and 2005 in the Bay of Fundy, Emerald Basin, and Roseway Basin, Canada, since these were the only long term recordings available for these areas that had previous well-known occurrence of NARWs. Data from a total of 324 recorders were analyzed, comprising 35,600 recording days of data. All government funded acoustic data are publicly available upon request from the data owner.

### Detection and classification of NARW calls

**Figure 6:**
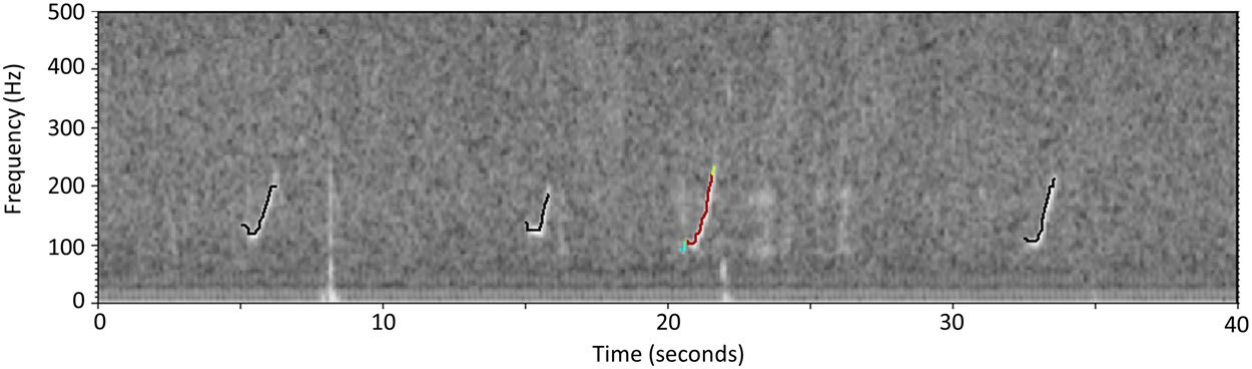
A spectrogram example produced by the Low Frequency Classification and Detection System, showing four North Atlantic right whale upcalls with their corresponding pitch tracks (black and colored lines). Warmer colors on the selected (colored) pitch track indicate high amplitudes of sound, while cooler colors indicate lower amplitudes.

All acoustic data were processed using the Low Frequency Detection and Classification System (LFDCS)^43^ which creates conditioned spectrograms (Fig. 6) using the short-time Fourier transform with a data frame of 512 samples and 75% overlap resulting in a time step of 64 ms and frequency resolution of 3.9 Hz. After tracing contour lines, or “pitch tracks”, through tonal sounds, the program uses multivariate discriminant analysis to classify the pitch tracks into call types. Calls were classified based on a user-developed call library; our library included four North Atlantic baleen whale species: NARW, fin (*Balaenoptera physalus*), sei (*B*. *borealis*), and humpback whales. Here, we focused only on the detections classified as NARW calls, specifically the low-frequency modulated upsweep known as the upcall. The upcall is a contact call used throughout the NARW range, produced by all ages and both sex classes, and is therefore the most reliable call to use for determining right whale presence^53,55^.

The call library described in Baumgartner and Mussoline^43^ was expanded and improved for this analysis to include a wider variety of examples of NARW upcalls and increase detection probability. Each detection was assigned a MD, which measures the deviation of a detection from the assigned call type (see Baumgartner and Mussoline^43^ for a more complete description). A lower MD indicates a closer match to the assigned call type. All NARW upcall detections with a MD less than or equal to 3.0 (after Baumgartner, *et al*.^63^) were manually screened by experienced analysts to determine which were correctly classified. For an ideal call type in the LFDCS (i.e., the seven attributes used in the discriminant function analysis are multivariate normal), 75% of actual calls will have a MD of 3.0 or less^63^; we chose this threshold to make the laborious process of manual screening manageable at the expense of sometimes missing genuine right whale upcalls (see below). This approach ensured that false detections were eliminated. The high degree of variability in NARW upcalls and the overlap with other species’ vocalizations, such as upsweeps produced by humpback whales, necessitated this extra manual step in data processing^63^.

For continuous data, a given day was marked as having NARWs present if three or more true upcall detections were found. Three upcalls were used to establish presence in order to be conservative and confident in stating NARW presence (we also conducted all analyses using a criterion of one upcall per day to indicate daily right whale presence, but neither our results nor conclusions changed). For duty-cycled data, the criteria was dropped to one true upcall detection signifying NARW presence so that presence was not underestimated due to a lower probability of recording vocalizing animals^64^. Weekly NARW acoustic presence per recorder was then summarized as the number of days per calendar week with daily presence.

To test whether the occurrence of right whales in regions differed over the two time periods, we ran a Generalized Linear Model (GLM) in R 3.4.1^65^, using the libraries *ggplot2* ^66^, *MASS* ^67^, *car* ^68^, and *phia* ^69^. This had the number of days in which whale calls were detected as the dependent variable, and the time periods (2004–2010; 2011–2014) and regions as independent variables, with their interaction effects included in the model. As the call data were counts, we ran the GLM with a Poisson distribution with log-link. The number of recording days was multiplied by the duty-cycle to correct for non-continuous data. Because effort (the number of days during which recorders were present) varied across time and region, we included the log of the number of days during which recorders were present plus 1 (as for some time*region cells, there were no recorders present) as an offset in the model.

The model formula in R was:

$$\begin{array}{c}{\rm{nDaysWithWhales}} \sim {\rm{timePeriod}}\ast {\rm{Region}},{\rm{family}}= \mbox{`} {\rm{poisson}}\mbox{'},\\ \,{\rm{offset}}=\,\mathrm{log}({\rm{nDaysRecordings}}+1)\end{array}$$

### Detector evaluation/missed detection rate

Detector performance was quantified to evaluate whether the missed detection rate for upcalls resulted in underestimating full days of NARW presence in our analysis. Three MARUs, manually reviewed for upcall presence in previous studies, were selected and used as ground-truth datasets to compare to our findings for days with NARW acoustic presence. These sites included year-round recordings from the Gulf of Maine (region 1); southern North Carolina (region 10); and Georgia (region 10)^21,48^. Selected days from each recorder were manually screened for NARW upcalls. Every third day of the Gulf of Maine recording was viewed and all upcalls for those days were counted (for detailed results on this analysis, see Bort, *et al*.^21^). Output from a detector^70^ run on North Carolina and South Carolina units was manually reviewed for daily presence of at least one NARW upcall (for detailed results on this analysis, see Hodge, *et al*.^48^). The resulting data were combined and used to generate a ground-truthed dataset of days with NARW presence; this was then compared to the number of days estimated to have NARW presence based on the manually screened output of the LFDCS system.

## Electronic supplementary material

Supplementary Table 1
