# Supplementary material for: Long-term passive acoustic recordings track the changing distribution of North Atlantic right whales (Eubalaena glacialis) from 2004 to 2014
Source: Sci Rep. 2017 Oct 18;7:13460. doi: 10.1038/s41598-017-13359-3 (PMC5647423; doi:10.1038/s41598-017-13359-3)
Supplement: Supplementary file 1 — Supplementary Table 1 [file 41598_2017_13359_MOESM1_ESM.pdf]

Long-term passive acoustic recordings track the changing distribution of North Atlantic right whales (*Eubalaena glacialis*) from 2004 to 2014

Genevieve E. Davis[1,2*], Mark F. Baumgartner[3], Julianne M. Bonnell[1], Joel Bell[4], Catherine Berchok[5], Jaqueline Bort Thornton[4], Solange Brault[2], Gary Buchanan[6], Russell A. Charif[7], Danielle Cholewiak[8], Christopher W. Clark[7], Peter Corkeron[8], Julien Delarue[9], Kathleen Dudzinski [10], Leila Hatch[11], John Hildebrand[12], Lynne Hodge[13], Holger Klinck[7, 14], Scott Kraus[15], Bruce Martin[9], David K. Mellinger[14], Hilary Moors-Murphy[16], Sharon Nieukirk[14], Doug Nowacek[13], Susan Parks[17], Andy Read[13], Aaron N. Rice[7], Denise Risch[18], Ana Širović [12], Melissa Soldevilla[19], Kate Stafford[20], Joy Stanistreet[13], Erin Summers[21], Sean Todd[22], Ann Warde[7], Sofie M. Van Parijs[8]

[1]Contracted under NOAA Northeast Fisheries Science Center, 166 Water Street, Woods Hole, MA 02543, USA
[2]University of Massachusetts Boston, 100 Morrissey Blvd, Boston, MA 02125, USA
[3]Woods Hole Oceanographic Institution, 266 Woods Hole Rd, Woods Hole, MA 02543, USA
[4]Naval Facilities Engineering Command Atlantic, Norfolk, VA 23508, USA
[5]NOAA Alaska Fisheries Science Center, 7600 Sand Point Way N.E., Seattle, WA 98115, USA
[6]New Jersey Department of Environmental Protection, Trenton, NJ 08625, USA
[7]Bioacoustics Research Program, Cornell Lab of Ornithology, Cornell University, 159 Sapsucker Woods Road, Ithaca, NY 14850, USA
[8]NOAA Northeast Fisheries Science Center, 166 Water Street, Woods Hole, MA 02543, USA
[9]JASCO Applied Sciences, 32 Troop Ave, Dartmouth, NS B3B 1Z1, Canada
[10]Dolphin Communication Project, Port St. Lucie, FL 34985, USA
[11]NOAA Stellwagen Bank National Marine Sanctuary, 175 Edward Foster Rd, Scituate, MA 02066, USA
[12]Scripps Institution of Oceanography, University of California San Diego, 9500 Gilman Dr MC 0205, La Jolla, CA 92037, USA
[13]Duke University Marine Laboratory, 135 Duke Marine Lab Rd, Beaufort, NC 28516, USA
[14]Oregon State University, 2030 SE Marine Science Drive, Newport, OR 97365, USA
[15]New England Aquarium, Central Wharf, Boston, MA 02110, USA
[16]Fisheries and Oceans Canada, Bedford Institute of Oceanography, 1 Challenger Drive, Dartmouth, NS B2Y 4A2, Canada
[17]Syracuse University, 107 College Place, Syracuse, NY 13244, USA
[18]The Scottish Association for Marine Science (SAMS), Oban, PA37 1QA, Scotland, UK
[19]NOAA Southeast Fisheries Science Center, 75 Virginia Beach Drive, Miami, FL 33149, USA
[20]University of Washington, Applied Physics Laboratory, 1013 NE 40th St, Seattle, WA 98105, USA
[21]Maine Department of Marine Resources, West Boothbay Harbor, ME 04575, USA
[22]Allied Whale, College of the Atlantic, 105 Eden St, Bar Harbor, ME 04609, USA
*genevieve.davis@noaa.gov

Supplementary Table 1: Data Summary: Summary of recorder locations, types, and configurations for all analyzed data. Recorders are grouped by deployment location with data gaps indicating periods of time within the recording dates that no recorders were in the water in that area.

Region	Location	Unit Type	# Units	Configuration	Recording Dates	Central Latitude	Central Longitude	Recording Schedule	Data Gaps
1	Davis Strait	HARU	3	Line	10/2006 - 10/2007	67.10	-57.72	continuous	
2	Iceland/Greenland	HARU	5	Array	05/2007 - 07/2008	60.90	-29.30	continuous	
	Emerald Bank, Scotian Shelf	HARU	2	Single	08/2005 - 06/2006	43.15	-63.67	continuous	
	Roseway Basin, Scotian Shelf	HARU	1	Single	08/2005 - 06/2006	42.97	-65.06	continuous	
	Eastern Scotian Slope	MARU	1-2	Single	07/2006 - 01/2007	43.78	-58.74	10/50 min on/off	09/2006 - 12/2006
	Eastern Scotian Slope	MARU	2-5	Single	08/2007 - 03/2009	43.99	-58.40	7/53 min on/off	10/2007 - 12/ 2007, 03/2008 - 06/2008, 09/2008 - 12/2008
	Eastern Scotian Slope	AMAR	1	Single	03/2010	43.95	-59.00	continuous	
	Gulf of St. Lawrence	MARU	2	Single	06/2010 - 09/2010	50.07	-63.92	continuous	
3	Eastern Scotian Slope	AMAR	3	Single	10/2012 - 09/2013	43.94	-58.52	13/2 min on/off	04/2013
	Emerald Basin, Scotian Shelf	Guardbuoy	1	Single	08/2013	43.37	-63.22	4.75/0.25 min on/off	
	Roseway Basin, Scotian Shelf	Guardbuoy	1-2	Single	08/2013 - 09/2013	42.90	-65.22	4.75/0.25 min on/off	
	Eastern Scotian Slope	AMAR	3	Single	11/2013 - 10/2014	43.95	-58.54	17/3 min on/off	04/2014
	Brown's Bank	MARU	1	Single	04/2014 - 09/2014	42.15	-65.39	continuous	
	Brown's Bank	HARU	1	Single	06/2014 - 10/2014	42.65	-64.15	continuous	
	Eastern Scotian Slope	AMAR	2	Single	07/2014 - 10/2014	43.60	-59.20	11.3/3.7 min on/off	
	Bay of Fundy	MARU	5	Array	08/2004, 08/2005	44.63	-66.45	continuous	
	Gulf of Maine	MARU	7	Single	07/2008 - 10/2008	44.02	-68.03	continuous	
	Gulf of Maine	MARU	1	Single	07/2009 - 10/2009	44.04	-68.07	continuous	
4	Gulf of Maine	MARU	1	Single	10/2009 - 10/2010	43.30	-68.62	continuous	06/2010
	Gulf of Maine	MARU	2	Single	07/2010 - 01/2011	43.08	-70.46	continuous	
	Gulf of Maine	MARU	9	Single	09/2010 - 12/2010	44.04	-68.57	continuous	
	Gulf of Maine	MARU	2	Single	10/2010 - 05/2011	43.37	-68.51	continuous	
	Gulf of Maine	MARU	1	Single	07/2011 - 09/2011	44.01	-68.07	continuous	
	Massachusetts Bay	MARU	1	Single	01/2006 - 05/2010	42.42	-70.28	continuous	06/2006, 06/2008
	Massachusetts Bay	MARU	3	Single	04/2011 - 05/2011	42.21	-70.17	continuous	
5	Massachusetts Bay	MARU	2	Single	08/2011 - 10/2011	42.20	-70.15	continuous	
	Massachusetts Bay	MARU	1	Single	06/2013 - 11/2013	42.30	-70.13	continuous	
	Massachusetts Bay	MARU	2	Single	11/2013 - 04/2014	42.25	-70.42	continuous	
	Massachusetts Bay	MARU	1	Single	10/2014 - 01/2015	42.40	-70.13	continuous	
	Nantucket Sound	AMAR	1	Single	10/2010 - 07/2011	41.50	-70.30	continuous	
	Georges Bank	MARU	3	Single	03/2012 - 06/2012	41.52	-68.87	continuous	
	Georges Bank	MARU	1	Single	03/2012 - 04/ 2012	42.09	-67.40	continuous	
	Georges Bank	MARU	1	Single	03/2012 - 09/2014	40.92	-66.520	continuous	07/2012 - 04/2014
6	Georges Bank	MARU	1	Single	03/2012 - 09/2014	41.36	-66.160	continuous	05/2012 - 04/2014
	Georges Bank	MARU	1	Single	05/2013 - 09/2014	40.23	-68.220	continuous	08/2013 - 04/2014
	Georges Bank	MARU	1	Single	04/2014 - 09/2014	40.58	-67.04	continuous	
	Georges Bank	HARU	1	Single	06/2014 - 01/2015	40.13	-66.25	continuous	
	Bear Seamount	AMAR	1	Single	07/2014 - 01/2015	40.29	-67.72	5.6/24.3 min on/off	
	New York	MARU	6-7	Line	02/2008 - 03/2009	40.32	-72.22	continuous	05/2008 - 8/2008
	Nantucket	MARU	5-8	Line	11/2011 - 02/2014	40.81	-70.52	continuous	10/2004 - 02/2013
7	Georges Bank	MARU	1	Single	05/2013 - 04/2017	40.65	-69.58	continuous	08/2013 - 04/2014
	Georges Bank	MARU	1-3	Single	05/2013 - 09/2014	39.93	-70.16	continuous	07/2013 - 04/ 2014
	Georges Bank	MARU	1	Single	04/2014 - 09/2014	39.49	-72.13	continuous	
	New York Harbor	MARU	2-3	Single	02/2008 - 03/2009	40.39	-73.67	continuous	05/2008 - 8/2008
	New Jersey	MARU	1-4	Single	03/2008 - 11/2009	39.44	-74.08	continuous	06/2009 - 08/2009
8	New Jersey	MARU	1-2	Single	06/2008 - 10/2009	39.42	-74.08	5/25 min on/off	06/2009 - 08/2009
	Delaware	AMAR	1	Single	06/2010 - 08/2011	38.70	-74.70	continuous	
	Virginia	MARU	4-5	Single	06/2012 - 07/2014	36.90	-75.26	continuous	
	Virginia	HARP	1	Single	06/2014 - 01/2015	37.17	-74.47	continuous	
9	Cape Hatteras	HARP	1	Single	03/2012 - 11/2014	35.34	-74.86	continuous	04/2012 - 10/2012, 03/2013 - 05/2014
	Cape Hatteras	MARU	4-5	Line	10/2013 - 01/2015	35.37	-75.16	continuous	06/2014 - 10/2014
	North Carolina	MARU	3	Line	01/2006 - 04/2006	33.61	-78.15	continuous	
	South Carolina	MARU	3	Line	01/2006 - 04/2006	32.66	-79.40	continuous	
	Georgia	MARU	3	Line	01/2006 - 04/2006	31.78	-80.84	continuous	
	North Carolina	HARP	1-2	Single	10/2007 - 06/2013	33.77	-76.29	5/5 min on/off	01/2008 - 05/2008, 09/2008 - 04/2009, 08/2009 - 07/2010, 03/2011 - 08/2011, 12/2011 - 07/2012
	North Carolina	HARP	1-2	Single	11/2009 - 04/2010	33.74	-76.50	5/10 min on/off	
	North Carolina	MARU	5	Array	07/2008	33.72	-76.53	continuous	
10	Florida	HARP	1-2	Single	04/2009 - 07/2011	30.27	-80.32	5/10 min on/off	12/1009 - 02/2010
	Florida	MARU	9	Array	09/2009 - 01/2010	30.20	-80.19	continuous	10/2009 - 12/2009
	Georgia	MARU	1	Single	11/2009 - 06/2011	31.83	-80.70	continuous	05/2010 - 01/2011
	Florida	MARU	1	Single	11/2009 - 05/2014	30.34	-81.21	continuous	05/2010 - 01/2011, 06/2011 - 12/2011, 05/2011 - 12/2012, 05/2013 - 11/2013
	Georgia	MARU	1	Single	06/2012 - 04/2013	31.86	-80.72	continuous	
	North Carolina	MARU	1	Single	06/2012 - 04/2013	34.17	-76.51	continuous	
	Georgia	MARU	1	Single	11/2012 - 05/2014	30.57	-81.23	continuous	04/2013 - 11/2013
	Florida	HARP	1	Single	05/2013 - 01/2015	30.27	-80.06	continuous	06/2013 - 02/2014
	Samana, Dominican Republic	MARU	4	Single	01/2009 - 03/2009	19.16	-69.20	continuous	
11	Saba Bank, Caribbean	MARU	1	Single	10/2011 - 04/28	17.51	-63.19	30/90 min on/off	
	Bermuda	HARP	1	Single	06/2013 - 03/2014	31.93	-65.15	continuous	
